# Allergic Inflammation Alters microRNA Expression Profile in Adipose Tissue in the Rat

**DOI:** 10.3390/genes11091034

**Published:** 2020-09-02

**Authors:** Dawid Szczepankiewicz, Wojciech Langwiński, Paweł Kołodziejski, Ewa Pruszyńska-Oszmałek, Maciej Sassek, Joanna Nowakowska, Agata Chmurzyńska, Krzysztof W. Nowak, Aleksandra Szczepankiewicz

**Affiliations:** 1 Department of Animal Physiology, Biochemistry and Biostructure, Poznań University of Life Sciences, 60-637 Poznań, Poland; dawszczepankiewicz@gmail.com (D.S.); pawel.kolodziejski@up.poznan.pl (P.K.); ewaprusz27@gmail.com (E.P.-O.); maciej.sassek@up.poznan.pl (M.S.); kwnowak@up.poznan.pl (K.W.N.); 2 Molecular and Cell Biology Unit, Department of Pediatric Pulmonology, Allergy and Clinical Immunology, Poznan University of Medical Sciences, 60-572 Poznań, Poland; wlangwinski654@gmail.com (W.L.); asianowakowska781@gmail.com (J.N.); 3 Institute of Human Nutrition and Dietetics, Poznań University of Life Sciences, 60-624 Poznań, Poland; agata.chmurzynska@up.poznan.pl

**Keywords:** inflammation, allergy, adipose tissue, lungs, miRNA

## Abstract

Adipose tissue is a major source of circulating exosomal microRNAs (miRNAs) that are modulators of the immune response in various types of tissues and organs, including airways. Still, no evidence exists if allergic airway inflammation may affect fat tissue inflammation via alterations in the miRNA expression profile. Therefore, we investigated the miRNA expression profile in the adipose tissue upon induced allergic inflammation in the airways in the rat. Brown Norway rats were chronically sensitized to house dust mite extract for seven weeks. Body composition was performed using MiniSpec Plus. The eosinophil count and the total IgE level were determined to confirm the induction of allergic inflammation. MiRNA expression profiling was done using the next-generation sequencing with validation by qPCR. We found that allergic airway inflammation significantly increased fat in adipose tissue, glucose concentration, and the gene expression of adipose tissue-derived proinflammatory peptides (leptin, TNFα). In miRNA-seq analysis, we showed significant differences in the expression of 36 mature miRNAs, three precursors, and two miRNA families in adipose tissue of allergic rats. Two miRNAs—miRNA-151-5p and miRNA-423-3p—showed significantly increased expression in qPCR in adipose tissue and lungs of sensitized animals. Allergic airway inflammation affects fat tissue and alters miRNA expression profile in adipose tissue in the rat.

## 1. Introduction

Adipose tissue is a major source of proinflammatory cytokines called adipokines. Recent studies suggest that adipose tissue-derived exosomal microRNAs (miRNAs) are a class of novel adipokines that may regulate metabolism in distant tissues, providing a new mechanism of cell-cell crosstalk [1]. Previous studies show that adipose tissue-derived miRNA levels correlated with several different conditions, including insulin insensitivity and diabetes [2,3,4,5], obesity [6], and cardiovascular disease [7].

Allergic airway inflammation is a hypersensitivity reaction to common aeroallergens (e.g., house dust mite) that underlies the pathogenesis of allergic rhinitis and asthma, two common respiratory diseases. As a result, upon allergen exposure, upper and lower airways are infiltrated by inflammatory cells, mainly Th2 lymphocytes and eosinophils [8,9] that activate inflammatory response (Th2 cytokines production, e.g., IL-4, IL-5, IL-13) and trigger IgE production and inflammatory mediators secretion (e.g., histamine). Chronically increased expression of inflammatory proteins further enhances inflammation and disease severity.

Adipose-derived miRNAs are also modulators of the immune response via immune cell and cytokine changes, thus linking adipose tissue with inflammation, including allergic inflammation [10]. Previous reports showed numerous miRNAs implicated in allergic diseases, including asthma (e.g., miR-21, miR-146a/b and miR-155, miR-19a, miR-24, miR-27) that regulate the secretion of Th1 and Th2 cytokines and promote differentiation of Th2 cells (as reviewed recently by Baskara-Yhuellou et al. and Specjalski et al. [11,12]). The recent studies showed that miRNAs also stimulate macrophage polarization into M1 inflammatory phenotype [13], activate mast cells [14], promote tolerogenic function of dendritic cells [15], and regulate airway remodeling in allergic asthma model [16].

To date, no studies investigated if miRNA expression profile in adipose tissue may undergo alterations upon allergic airway inflammation and if these changes affect miRNA expression in the lungs. Recent work demonstrated in the adipose tissue-specific knockout of Dicer that this tissue is a major source of circulating exosomal miRNAs in mice and may regulate whole-body metabolism and mRNA translation in other tissues [1]. Therefore, we assumed that allergic airway inflammation affects fat tissue via alterations in the miRNA expression profile in adipose tissue. To test this assumption, we investigated the miRNA expression profile in the adipose tissue upon allergic inflammation in the airways and if it reflects alterations in fat tissue of sensitized rats.

## 2. Materials and Methods

### 2.1. Animal Model of Allergic Inflammation

In the study, we used brown Norway male rats to model allergic inflammation induced by house dust mite (HDM) inhalation as previously described [17] with minor modifications. The study was approved by the local ethical committee (agreement no. 35/2017).

Twenty-three male brown Norway rats purchased from Janvier Labs (Le Genest-Saint-Isle, France) with a baseline weight of 170 g ± 15 g. The animals were housed and kept for one week of acclimatization under standard conditions. In all rats, weight, body composition analysis, blood glucose, and serum IgE were measured. Then, the animals were randomly allocated into two experimental groups: sensitized (*n* = 10) and control (*n* = 13) group. The body mass of rats was measured every week on the same day. Body composition, including total body fat content, was performed using MiniSpec Plus (Bruker, Rheinstetten, Germany) at the baseline and after sensitization protocol. The experiments were conducted during the light phase at approximately 09:00.

**Figure 1:**
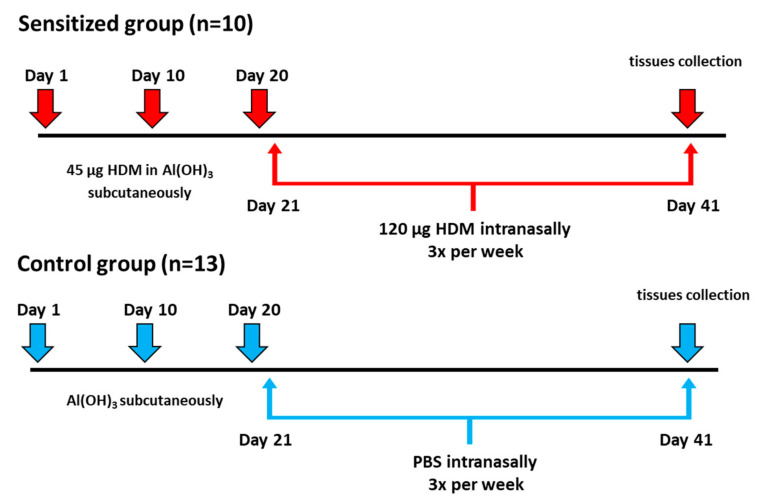
The experimental design of allergic sensitization protocol in brown Norway rats.

Rats from the sensitized group were treated by subcutaneous injection of 250 µL HDM extract (45 µg, Citeq Biologics, Groningen, The Netherlands) in 4% Al(OH)3 (Thermo Fisher Scientific, Foster City, CA, USA) as an adjuvant. Control rats were injected with an equivalent volume of adjuvant. Injections were done once a week for three weeks. After that time, animals from the asthmatic group were receiving intranasally HDM extract (120 µg/50 µL three times per week for four weeks). Rats from the control group were intranasally exposed to the same volume of PBS with the corresponding experimental schedule. The experimental design was shown od Figure 1.

After that, rats were sacrificed by decapitation. Bronchoalveolar lavage (BALF), blood for serum, lungs, and epididymal adipose tissue were immediately collected. All samples were collected at the same time (09:00 after overnight fasting). Blood vessels, if any, were removed from adipose tissue before storage. Tissues were snap-frozen in liquid nitrogen, and all samples were stored at −80 °C before miRNA extraction.

### 2.2. Histopathological Analysis

After cleaning the lungs from blood, they were fixed in 10% formalin in saline for 72 h and embedded in paraffin. Paraffin sections (5 μm) were stained with hematoxylin and eosin (H&E).

### 2.3. Eosinophil Count

The percentage of eosinophils was counted in whole blood smears prepared manually by the wedge-spread film technique, using the May–Grünwald and Giemsa stains. The eosinophil count was done twice after two and four weeks of intranasal exposition of HDM and defined as the number of eosinophils per 100 counted blood cells for each sample.

### 2.4. Glucose Measurement

Glucose blood level was measured by AccuCheck glucometer (Roche, Warsaw, Poland) from tail vein 12 h after food deprivation. The measurement was done after acclimatization before the sensitization experiment and one day before decapitation.

### 2.5. ELISA Method

Total IgE level was evaluated using ELISA kit (ElabScience, Wuhan, Hubei, China) in 10-fold diluted serum samples for each rat according to the manufacturer’s protocol. All samples were run in duplicates. The absorbance was read on a plate reader at 450 nm wavelength (Asys UVM 340, Biogenet, Otwock, Poland). Protein concentration was quantified against a standard curve calibrated with known amounts of protein. The detection range of the kit was 1.56–100 ng/mL. The intra- and inter-assay variability was about 5%.

### 2.6. Small RNA Sequencing

RNA and miRNA from epididymal adipose tissue (six asthmatic and sic control rats) were extracted using ExtractMe miRNA kit (Blirt, Gdansk, Poland). The quantity of miRNA samples was measured using microRNA assay kit (ThermoFisher Scientific, Foster City, CA, USA). The miRNA expression profile from epididymal adipose tissue and lung tissue was performed using next-generation sequencing. Libraries were generated from 50 ng of miRNA sample using TruSeq small RNA library preparation kit (Illumina, San Diego, CA, USA) following the manufacturer’s instructions. After size selection in polyacrylamide 8% gel electrophoresis, libraries were validated and quantified using High Sensitivity DNA Screen Tape (Agilent, Santa Clara, CA, USA) on Tape Station 2200 (Agilent) and run on MiniSeq sequencer (Illumina, San Diego, CA, USA) with 50 nt single-end reads. Differential miRNA expression analysis was done in Base Space software (DeSeq algorithm, Illumina, San Diego, CA, USA), and reads were mapped to the rat genome (miRbase v21). Raw sequencing data were stored in NCBI SRA database (http://www.ncbi.nlm.nih.gov/bioproject/657964).

### 2.7. MiRNA Expression Analysis

For validation by qPCR, we selected two miRNA genes that showed the highest fold change and the lowest *p* value in differential gene expression analysis in miRNA-seq between sensitized and control rats in adipose tissue and lung tissue (10 asthmatic and 13 control rats). The expression of the same miRNA genes was also examined in BALF-derived exosomes from these animals (*n* = 23). Exosomes from BALF were precipitated using miRCURY Exosome Isolation Kit (Qiagen, Wroclaw, Poland), and RNA and microRNA from BALF-derived exosomes were extracted using RNeasy mini kit (Qiagen). For reverse transcription, we used MystiCq microRNA cDNA Synthesis Mix (Sigma-Aldrich, Darmstadt, Germany. Quantitative PCR was done using MystiCq microRNA SYBR Green qPCR ReadyMix (Sigma-Aldrich, Darmstadt, Germany) and microRNA assays for miR-151-5p, miR-423-3p, and RNU-6 (endogenous control). Differential expression results from qPCR were compared using Data Assist software (ThermoFisher Scientific, freely available form website) using the relative quantification method.

### 2.8. Gene Expression Analysis

Total RNA was isolated from adipocytes with NucleoSpin RNA (MACHEREY-NAGEL, Dueren, Germany) according to the manufacturer’s protocol. The amount and quality of isolated RNA was measured on spectrophotometer NanoDrop 1000 (Thermo Scientific, Wilmington, DE, USA). Total RNA (1 µg) was used for reverse transcription with FIREScript RT cDNA Synthesis MIX with Oligo (dT) and random primers (Solis BioDyne). A negative control (RT-) lacking reverse transcriptase was also prepared. Resulting cDNA was used for quantitative RT-PCR (qPCR) using 5x HOT FIREPol EvaGreen qPCR Mix Plus (ROX) (Solis BioDyne) and specific primers for leptin, insulin receptor and glucose transporter, interleukin 6 and TNFα [18]. PCR was run on Quant Studio 12K Flex system (Thermo Fisher Scientific, USA). The expression was analyzed using the comparative ∆∆Ct method. Melting curve analysis was done to verify the specificity of PCR product. For each sample, the expression levels of the target genes were normalized to the reference gene (*Gapdh*). Negative and positive control of reverse transcription and qPCR reactions were also performed.

### 2.9. MiRNA Pathway Analysis

The significantly altered expression of 36 mature miRNAs was used for pathway analysis in the *Database* for Annotation, Visualization and Integrated Discovery (DAVID). For the target prediction of miRNAs down- and upregulated in our miRNA profiling, we used miRGate database available at http://mirgate.bioinfo.cnio.es/miRGate using several computational prediction algorithms (Miranda, Pita, Microtar, Targetscan) and validated databases (Mirtarbase, Mirecords, Tarbase). For pathway analysis in DAVID, we included targets predicted by at least three computational algorithms or confirmed by a validated database (experimentally validated targets). From DAVID, we selected only those pathways with *p* < 0.05 after the false discovery rate (FDR) adjustment and EASE score below 0.05 (significantly enriched in the annotation categories).

### 2.10. Statistical Analysis

The normal distribution of the data was checked with the Shapiro–Wilk test. The data that compared fat tissue parameters (weight, % of fat, glucose level), mRNA expression levels (*Lep, Insr*, and *Slca2a4*), IgE levels, and eosinophil count and miRNA expression (miR-151-5p and miR-423-3p) in adipose tissue, lungs, and BALF between sensitized (*n* = 10) and control rats (*n* = 13) were analyzed using t-test the in Graph Pad Prism software (GraphPad Software Inc., San Diego, CA, USA). All tests were two-tailed. The α significance level for all tests was below 0.05.

## 3. Results

### 3.1. Metabolic and Inflammatory Parameters in An Allergy Model

**Figure 2:**
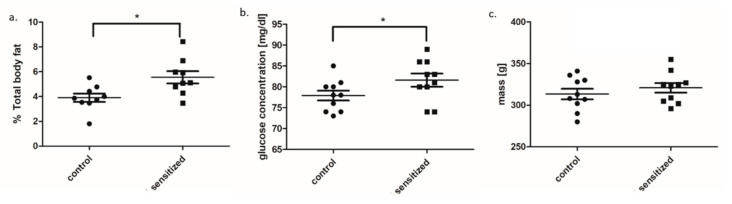
The comparison of metabolic parameters (**a**) % fat, (**b**) glucose level, and (**c**) weight between sensitized and control rats (dot plot, mean ± standard deviation, * *p* < 0.05).

**Figure 3:**
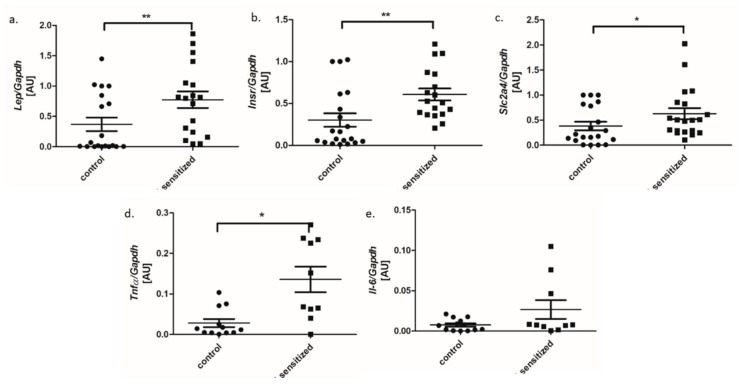
The expression of mRNA genes involved in adipose tissue metabolism: (**a**) leptin, (**b**) insulin receptor and (**c**) Slc2a4, (**d**) TNF-α, and (**e**) IL-6 in adipose tissue of sensitized and control rats (dot plot, mean ± standard deviation, * *p* < 0.05; ** *p* < 0.01). AU—arbitrary units.

Fat amount expressed as % of total body fat significantly increased in the sensitized rats in comparison to control rats (Figure 2a). We also observed elevated mean serum glucose concentration in sensitized rats (Figure 2b). The mean weight was slightly increased in the allergic group but did not differ significantly from the control rats (Figure 2c). Analysis of genes related to adipose tissue metabolism showed significantly increased mRNA expression of leptin (*Lep*), insulin receptor (*Insr*), and glucose transporter (*Slca24*) in sensitized animals as compared to the control group (Figure 3a–c). Analysis of pro-inflammatory genes showed increased expression TNF-α and IL-6 (FC = 2.7 and FC = 3.5, respectively) in adipose tissue in sensitized rats as compared to the control group (Figure 3d,e).

**Figure 4:**
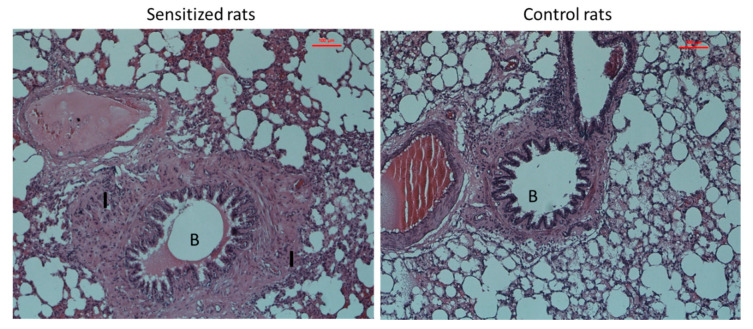
The comparison of histological changes in the lung tissue between sensitized (**on the left**) and control rats (**on the right**). Photomicrographs of lung sections from experimental groups (hematoxylin and eosin (H&E) × 400). The sensitized group shows the distortion of the typical bronchiolar architecture (letter B), peribronchiolar and alveolar inflammatory cellular infiltrates (letter I). The control group shows the normal bronchiolar architecture, including healthy epithelial lining and thin, smooth muscle layer (letter B).

**Figure 5:**
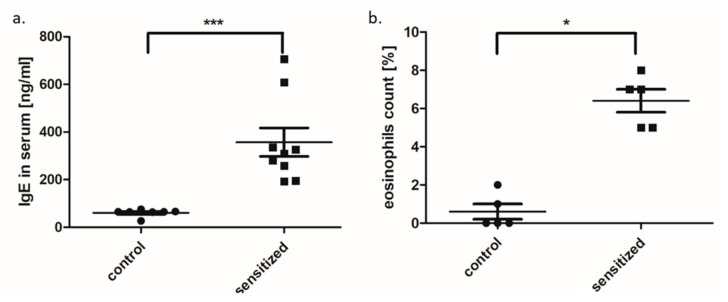
The comparison of total IgE level (**a**) and eosinophil counts (**b**) between sensitized and control rats (dot plot, mean ± standard deviation, * *p* < 0.05, *** *p* < 0.001).

Lung histology and blood analysis (eosinophil count and the concentration of total IgE) confirmed the presence of allergic inflammation in the lungs as well as in the peripheral blood of sensitized animals. The presence of inflammation in the airways was confirmed by histological analysis–hypertrophic muscles, obstructed airways, mucus overproduction (Figure 4). Peripheral blood analysis showed significantly elevated eosinophil counts and increased concentration of total IgE in the serum of sensitized group as compared to the control rats (*p* = 0.012 and *p* < 0.001, respectively) (Figure 5).

### 3.2. Small RNA Expression Profile in Adipose Tissue

**Figure 6:**
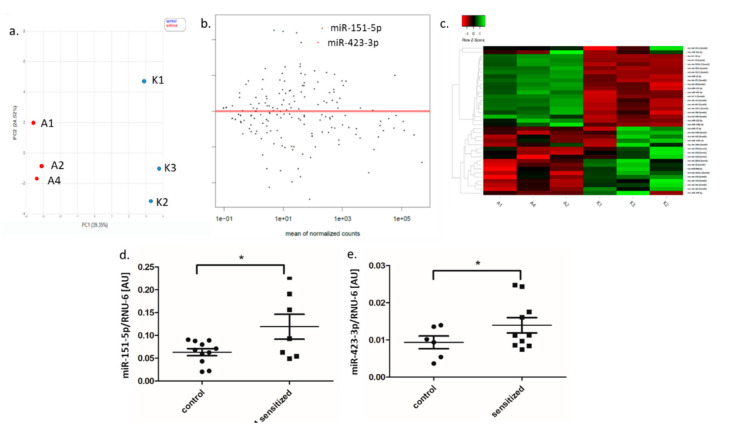
Differential expression analysis of significantly altered miRNA expression in adipose tissue between sensitized and control rats; miRNA-seq analysis: (**a**) PCA plot of control (C) and sensitized (A) rats; (**b**) MA plot and (**c**) heat map of differentially expressed miRNAs; results of qPCR validation: (**d**) miR-151-5p and (**e**) miR-423-3p in adipose tissue (dot-plot, mean ± standard deviation, * *p* < 0.05). PCA–principal component analysis, MA plot–visualizes the differences between groups, by transforming the data onto M (log ratio) and A (mean average) scales; AU—arbitrary units.

Genome-wide sequencing of miRNA expression profile showed that in adipose tissue, 1689 mature miRNAs and isomiRs underwent expression, and 536 miRNAs passed the low-count filter (10 reads). We observed a significantly increased expression of 16 and a decreased expression of 20 mature miRNAs and isomiRs in sensitized rats as compared to the control group (Table 1, Figure 6a–c). From these, we selected two miRNAs, miR-151-5p and miR-423-3p, that had the highest fold change and the lowest *p* value for qPCR validation. Their expression was significantly higher in sensitized rats as compared to the controls (Figure 6d,e). In the miRNA precursors, 311 underwent expression in adipose tissue, and 146 passed the low-count filter (10 reads). Differential expression analysis showed altered expression for three precursors between sensitized rats and the control group: rno-miR-423 and rno-let-7d were upregulated, and rno-miR-99a was downregulated.

Analysis of miRNA family distribution between sensitized and control animals showed 198 expressed miRNA families. From these, 99 passed the low-count filter, and two (miR-423 and miR-335) showed a significant increase in expression in asthma as compared to the control group.

Sequencing of small RNA also showed 318 piRNAs expressed in adipose tissue with 46 passing low-quality filter. None of these was differentially expressed in asthma as compared to control adipose tissue samples.

### 3.3. MiRNA Expression in Lung and BALF

**Figure 7:**
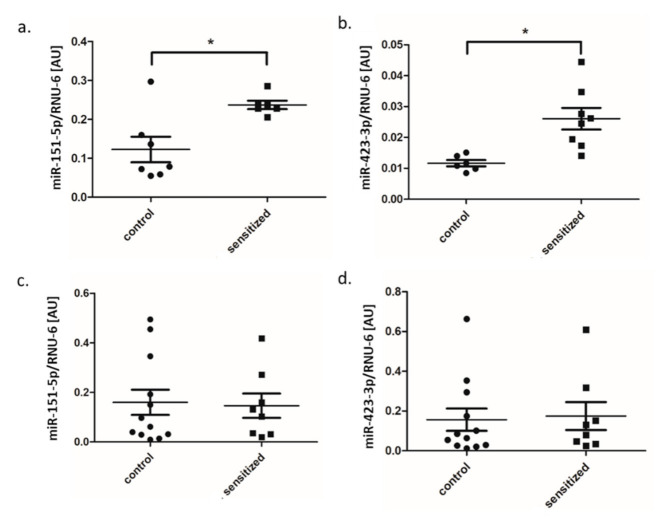
Relative expression of (**a**) miR-151-5p and (**b**) miR-423-3p in lung tissue; the expression of (**c**) miR-151-5p and (**d**) miR-423-3p in BALF (dot plot, mean ± standard deviation, * *p* < 0.05). BALF—bronchoalveolar lavage, AU—arbitrary units.

Based on the miRNA sequencing results, we selected two miRNAs (miR-151-5p, miR-423-3p) that showed significantly upregulated expression in adipose tissue of sensitized rats for both canonical form and isomiR for qPCR validation in the lung tissue (*n* = 13) and BALF (*n* = 13). In sensitized rat lungs, we confirmed by qPCR significant increase in the expression of miR-151-5p and miR-423-3p as compared to the control group (*p* = 0.013 and *p* = 0.046, respectively) (Figure 7a,b). In BALF-derived extracellular vesicles, we found no difference in the expression of these two miRNAs by qPCR in sensitized rats as compared to the control group (*p* > 0.05) (Figure 7c,d).

### 3.4. MiRNA Pathway Prediction

Pathway analysis of target genes for upregulated miRNAs showed 11 significantly enriched pathways, including e.g., TGF-β signaling pathway, FoxO signaling pathway, leukocyte transendothelial migration, bacterial invasion of epithelial cells (Table 2).

For downregulated miRNAs targets, none of the biological pathways showed significant enrichment in adipose tissue of sensitized rats. However, seven gene ontology terms showed substantial enrichment for targets of downregulated miRNAs (Table 3).

## 4. Discussion

The main finding of this study is that allergic airway inflammation affects fat tissue that is accompanied by altered miRNA expression profile in adipose tissue. Two of miRNAs increased in adipose tissue also showed an increase in the lungs of sensitized rats suggesting their involvement in inflammatory processes in both adipose tissue and the airways. Apart from altered mature miRNA and isomiRs, we also observed changes in the expression of miRNA precursors and miRNA families in adipose tissue of allergic rats. This observation suggests that airway inflammation affects mature miRNA expression and post-transcriptional regulation of miRNA precursors and miRNA co-localized genes (i.e., families).

The previous studies focused on the effect of fat tissue and obesity on allergic airway inflammation [19,20,21,22], but the impact of airway inflammation on fat tissue was not analyzed to date. The observation that allergic inflammation influences the fat tissue is consistent with our previous report [18] where we showed a stimulating effect of IL-4, the main Th2-cytokine, on lipogenesis in mature rat adipocytes. Enhanced lipogenesis increased fat accumulation and significantly altered adipocyte metabolism. Our observation that fat tissue metabolism alterations are accompanied by a change in miRNA expression profile, led us to the assumption that miRNAs play a role in this process.

Previous studies reported altered expression of several miRNA involved in immune response and allergic inflammation in target tissues (lungs, nose, skin) as reviewed recently [23,24]. However, our findings are the first to show that changes during allergic airway inflammation also affect miRNA expression in adipose tissue, thus influencing its metabolism. Sixteen upregulated and 20 downregulated mature miRNAs, and isomiRs in sensitized rats regulate several pathways previously linked to allergic inflammation or airway hyperresponsiveness including TGF-β signaling pathway [25,26], FoxO signaling pathway [27], leukocyte transendothelial migration [28], and bacterial invasion of epithelial cells [29].

We also showed that two miRNAs upregulated in adipose tissue, miR-423-3p, and miR-151-5p showed analogous changes in the lungs of sensitized rats. A previous study in the ischemic-reperfusion model indicated that miR-423-3p regulates ERK signaling pathway, a key regulator of ER-induced apoptosis and ischemia-reperfusion injury. Increased expression of this miRNA resulted in decreased ERK expression, and miR-423-3p/ERK signaling pathway reduced ER-induces stress and myocardial apoptosis [30]. MiR-423-3p overexpression also promoted cell proliferation, migration, and invasion of lung cancer cells [31,32]. Its increased plasma level correlated with total fat loss in patients with diabetes type 2 after four months of training [33]. The authors also reported that miR-423-3p targeted genes involved in fatty acid metabolism and biosynthesis pathways. The role of this miRNA in regulating lipid metabolism was shown in rats fed with a high-fat diet that resulted in increased expression of miR-423-3p and correlated inversely with the expression of apolipoprotein D [34]. Thus, this miRNA seems a regulator of fat tissue metabolism during allergic inflammation.

Previous findings showed that mir-151-5p overexpression inhibited IL4Rα/mTOR pathway and decreased IL4/IL4Rα signaling. This enhanced osteogenic differentiation and reduced adipogenic differentiation in the mouse model of systemic sclerosis [35]. Moreover, miR-151-5p was upregulated in the lungs infected with influenza mice treated with anti-miR-151-5p inhibitor regained body weight and survived influenza infection [36]. These reports suggest that this miRNA may be involved not only in adipose tissue metabolism but also in inflammatory response regulation.

Despite mature miRNA, also precursors showed differential expression in adipose tissue of sensitized rats: rno-miR-423 and rno-let-7d were upregulated, and rno-miR-99a was downregulated. Increased expression of miR-423 precursor was previously reported in Rho/Rac signaling pathway [37] involved e.g., in airway smooth muscle contraction in asthma [29]. Recent studies showed that Rho GTPases are regulators of glucose homeostasis responsible for glucose uptake by adipose tissue [38]. The upregulation of let-7adf cluster was observed in lipopolysaccharide-activated macrophages and promoted IL-6 secretion by down-regulating Tet2 [39]. Therefore, increased expression of let-7d found in adipose tissue of allergic rats in our study is in line with the previous observations of increased IL-6 production in allergic inflammation [40,41]. Subcutaneous adipose tissue overexpressed let-7d also in HIV-infected patients with lipodystrophy syndrome [42]. A precursor of miR-99a was previously involved in apoptosis in cancer research [43,44], and its overexpression inversely correlated with mTOR expression enhancing apoptosis. In the study by Guo et al., miR-99a increased during adipogenesis [45], developmental growth [46], and adipocyte maturation [42].

Taking into account previous studies reporting that altered post-transcriptional regulation of miRNA precursors by RNA binding proteins may promote cancer [47,48,49,50], observed changes in the miRNA precursors expression between sensitized and control animals may indicate their role in allergic inflammation. However, further functional studies are warranted to verify this hypothesis.

In our study, we also found altered expression of two miRNA families, suggesting that also whole sets of genes with common conserved sequence and similar function [51] undergo changes upon allergic inflammation in adipose tissue. A previous study showed that miRNA genes from the same family are non-randomly co-localized and organized around genes involved in the immune system [52], indicating that co-localized miRNA genes from the same family may be similarly regulated. In our study, two miRNA families showed upregulation in sensitized rats, miR-423, and miR-335. Previous studies showed that, in obese mice fed with a high-fat diet, miR-335 was upregulated by TNFα in adipose tissue leading to decreased expression of genes regulating insulin signaling and lipid metabolism [53]. In humans, this miRNA increased leptin, resistin, TNFα, and IL-6 levels in mature adipocytes and it was upregulated during human adipocyte differentiation [54]. The authors also showed that high-fat diet induced obesity resulted in systemic low-grade inflammation and increased proinflammatory cytokines expression in plasma (i.e., TNF-α, IFN-γ, IL-6, MCP-1β, IL-1, and IL-17). Therefore, the miR-335 family may be a link between allergic inflammation and impaired metabolism in adipose tissue.

In the PCR validation step, we found that two miRNAs (miR-151-5p and miR-423-3p) overexpressed in adipose tissue were also upregulated in the lungs, but not in BALF. The possible explanation of observed differences may be that these two different airway compartments demonstrate different miRNA expression patterns. A similar result was previously observed by Conickx et al. [55]. They found a clear separation between miRNA profiles from BALF and lung tissue, indicating that these two compartments have different cellular identity, anatomical structure, and organization [55].

The strength of our study is the use of a tool that integrates prediction algorithms and databases with functionally validated targets for pathway analysis. This combination decreases the probability of random discoveries and increases the reliability of our pathway analyses. The main limitation of the study, however, is a lack of functional experiments that could elucidate the role of these miRNAs in regulating adipose tissue metabolism during allergic inflammation.

## 5. Conclusions

MiRNA expression profile changes in the adipose tissue upon allergic airway inflammation in the rat model and includes not only mature miRNAs but also precursors and miRNA families. Pathways regulated by these altered miRNAs influence not only inflammatory responses but also adipose tissue metabolism. However, further functional studies are warranted to verify these observations.

## Figures and Tables

**Table 1:** The list of down- and upregulated miRNAs in adipose tissue of sensitized rats (DesSeq).

miRNA	Control	Sensitized	log2FC	p corr
Downregulated				
rno-mir-30a	497.871	135.472	−1.878	0.000
rno-mir-181d	17.772	5.331	−1.737	0.003
rno-mir-21	313.798	96.820	−1.696	0.000
rno-mir-30e	51.538	17.866	−1.528	0.001
rno-miR-194-5p	79.663	28.303	−1.493	0.022
rno-mir-181b-2	42.018	15.548	−1.434	0.003
rno-mir-140	224.135	83.272	−1.428	0.000
rno-mir-140 (isomiR)	16.408	6.139	−1.418	0.035
rno-miR-99a-5p	2355.335	920.727	−1.355	0.000
rno-mir-140 (isomiR)	71.921	31.032	−1.213	0.024
rno-mir-10b (isomiR)	8594.443	3779.265	−1.185	0.000
rno-mir-10b (isomiR)	5186.707	2673.096	−0.956	0.002
rno-mir-10b (isomiR)	17436.284	9038.696	−0.948	0.000
rno-mir-10b	389984.419	207561.930	−0.910	0.002
rno-miR-25-3p	1095.843	609.184	−0.847	0.001
rno-miR-203b-3p	404.280	226.100	−0.838	0.003
rno-mir-148a	1710.533	985.831	−0.795	0.019
rno-mir-101a	592.118	383.474	−0.627	0.042
rno-miR-30a-5p	1980.284	1332.122	−0.572	0.032
rno-miR-148a-3p	848.973	632.695	−0.424	0.044
Upregulated				
rno-miR-22-3p	42064.320	61447.574	0.547	0.047
rno-mir-26a	1845.464	3083.088	0.740	0.013
rno-mir-203a	80.248	134.653	0.747	0.025
rno-miR-151-5p	330.363	559.598	0.760	0.005
rno-let-7e	212.724	382.057	0.845	0.001
rno-mir-125b-2	59.775	107.911	0.852	0.011
rno-let-7d-5p	198.981	361.600	0.862	0.000
rno-mir-186	164.726	310.721	0.916	0.044
rno-mir-28	33.449	65.084	0.960	0.034
rno-miR-365-3p	21.838	44.120	1.015	0.045
rno-let-7d	121.531	248.091	1.030	0.000
rno-mir-151 (isomiR)	63.952	139.470	1.125	0.000
rno-mir-423 (isomiR)	39.236	91.327	1.219	0.001
rno-miR-423-3p	31.664	76.350	1.270	0.011
rno-mir-322-1	15.317	40.091	1.388	0.004
rno-mir-322-2	11.173	36.720	1.717	0.000

**Table 2:** DAVID pathway analysis results for targets of miRNAs upregulated in adipose tissue of sensitized rats.

Term	Gene Count	Fold Enrichment	*p* corr	Genes
Mineral absorption	7	6.293	0.001	*FTL1, SLC5A1, HMOX1, SLC26A9, MT1, SLC39A4, FTH1*
Lysosome	11	3.037	0.003	*ATP6V0C, AP1S1, CLTB, HEXA, NAGA, GALC, ABCA2, CD164, ATP6V0A4, AP4S1, AP3B1*
Bacterial invasion of epithelial cells	8	3.463	0.008	*ACTB, CAV3, CBLB, CLTB, HCLS1, PIK3R5, ARPC4, PXN*
TGF-β signaling pathway	8	3.262	0.011	*MAPK1, ACVR1B, ROCK1, TGFBR1, ZFYVE16, ZFYVE9, PITX2, ACVR1*
Tuberculosis	12	2.312	0.014	*ATP6V0C, CIITA, MAPK1, CYP27B1, PPP3CB, CALM3, FCGR2A, ATP6V0A4, RFXANK, IFNGR1, CD74, IL10*
Osteoclast differentiation	9	2.446	0.030	*MAP3K7, MAPK1, TGFBR1, PPP3CB, PIK3R5, FCGR2A, FOSL1, IFNGR1, CSF1R*
Synaptic vesicle cycle	6	3.393	0.031	*ATP6V0C, CLTB, CPLX1, AP2S1, ATP6V1G1, ATP6V0A4*
Dorso-ventral axis formation	4	5.610	0.033	*NOTCH3, MAPK1, ETS2, NOTCH4*
FoxO signaling pathway	9	2.320	0.039	*CCNB1, MAPK1, PRMT1, RBL2, ATG12, TGFBR1, PIK3R5, STAT3, IL10*
Endocytosis	14	1.818	0.044	*CAV3, ARFGAP2, CLTB, USP8, AP2S1, TGFBR1, ARPC4, CBLB, DAB2, ZFYVE16, ZFYVE9, SPG21, BIN1, VPS25*
Leukocyte transendothelial migration	8	2.397	0.048	*ACTB, RASSF5, ROCK1, PTK2B, CLDN1, PIK3R5, PXN, CLDN23*

DAVID: *Database* for Annotation, Visualization and Integrated Discovery.

**Table 3:** DAVID GO terms analysis for targets of miRNAs downregulated in adipose tissue in sensitized rats.

Term	Gene Count	*p* corr	Fold Enrichment	Genes
GO:0030100~regulation of endocytosis	3	0.007	24.021	*ARFGAP1*, *SYT4*, *RAB4B*
GO:0021831~embryonic olfactory bulb interneuron precursor migration	2	0.008	240.205	*ARX*, *RAC1*
GO:0043652~engulfment of apoptotic cell	2	0.020	96.082	*BECN1*, *RAC1*
GO:0046929~negative regulation of neurotransmitter secretion	2	0.020	96.082	*SYT4*, *PNKD*
GO:0006366~transcription from RNA polymerase II promoter	6	0.024	3.621	*HAND1*, *CCNH*, *GTF2H4*, *NEUROD1*, *DDX21*, *RUNX2*
GO:0032471~negative regulation of endoplasmic reticulum calcium ion concentration	2	0.032	60.051	*BAK1*, *ATP2A1*
GO:0014049~positive regulation of glutamate secretion	2	0.048	40.034	*SYT4*, *ADORA2A*
